# Correlations between pain, functioning, and quality of life in manual wheelchair users with spinal cord injury

**DOI:** 10.1371/journal.pone.0324850

**Published:** 2025-05-29

**Authors:** Mokgadi Kholofelo Mashola, Elzette Korkie, Diphale Joyce Mothabeng

**Affiliations:** 1 Department of Physiotherapy, School of Therapeutic Sciences, Faculty of Health Sciences, University of the Witwatersrand, South Africa; 2 Department of Physiotherapy, School of Healthcare Sciences, Faculty of Health Sciences, University of Pretoria, South Africa; University of Illinois Urbana-Champaign, UNITED STATES OF AMERICA

## Abstract

**Background:**

Pain after spinal cord injury (SCI) is one of the most important contributors to poor rehabilitation outcomes, reduced quality of life (QOL) as well as poorer physical, social, and psychological functioning.

**Objective:**

To determine the correlation of overall and shoulder pain on functioning and QOL in community-dwelling people with SCI.

**Methods:**

This quantitative correlational study included people with SCI with or without pain, who were discharged from five rehabilitation hospitals in Gauteng, South Africa. The presence of pain, wheelchair function, and QOL were investigated using the Numeric Rating Scale, Wheelchair Function Test Questionnaire, and the WHOQOL-BREF questionnaire. Pectoralis minor muscle (PMm) length was measured using a Vernier caliper and the Scapular Dyskinesis test was used to observe for scapular dyskinesis. Descriptive statistics; Independent t-tests, ANOVA tests, and Fisher’s exact tests were performed using the SPSS v27 at a 0.05 level of significance.

**Results:**

85% of the 122 participants reported overall pain, mainly burning (32.7%) and below the level of injury (39.4%), with only 14.8% reporting shoulder pain. There was no overall difference in QOL between participants with and without pain, however, pain prevented individuals from doing what they needed to do (*p* < 0.05). Pain severity was negatively correlated with QOL (*p* < 0.001) and sleep satisfaction (*p* < 0.05). There were no correlations found between shoulder pain and PMm length, as well as scapular dyskinesis. However, there was a negative correlation between shoulder pain and wheelchair function in those who reported shoulder pain (*p* < 0.01).

**Conclusion:**

Pain is problematic after SCI and although shoulder pain is not as prominent, it has the potential to negatively impact an individual’s ability to use their wheelchair effectively.

**Contribution:**

Findings from this study emphasise the negative role of pain on life satisfaction and QOL. Stakeholders involved in SCI rehabilitation can consider including comprehensive pain management within the interprofessional model of care.

## Introduction

Pain is a commonly prevalent and significant health problem in both the general and spinal cord injury (SCI) population. Up to 94% of people with spinal cord injury (PWSCI) experience nociceptive and/or neuropathic pain, and at least one-third will have severe pain [1–3]. Pain after SCI tends to be chronic, occurring for more than three months, and associated with emotional distress and depression, and the higher the severity of pain, the worse the negative impact on an individual [1]. People with SCI who report severe pain levels may refrain from fully engaging in their community due to the pain levels, leading to diminished enjoyment, productivity, and strained relationships, which negatively impact their quality of life (QOL) [4,5]. The presence of pain has been shown to interfere with mobility functioning and ADLs, and in addition, directly contributes to further functional disability as it reduces the affected individual’s capacity to return to work, and subsequently plays a pivotal role in creating financial problems [3]. It is understandable how the pain experience then cascades into a worsened cognitive and emotional function (such as mood and sleep disorders, emotional distress, and depression); leading to a significant impact on self-perception of health and QOL [1,6]. This may lead to an increased risk of developing secondary health conditions (SHCs) and increasing healthcare costs associated with general SHC management and hospital readmission [7].

The dynamic interaction between demographic information, SCI profile, psychological status, and social factors highlights the multidimensionality of pain, making the pain experience unique and individualized [8]. For manual wheelchair users, scapula performance is crucial in effective shoulder stability, position, movement, and muscle performance during wheelchair activities [9]. A decreased pectoralis minor muscle (PMm) length due to scapular dyskinesis (which is altered scapular motion) or adaptive muscle shortening has been identified to play a role in shoulder dysfunction [10]. People with SCI spend an estimated 8.3 hours per day seated in their wheelchair performing manual daily tasks in 30˚ of forward flexion and performing 14–18 transfers a day [11]. This may explain why manual wheelchair users generally present with a round-shoulder posture, which is characterised by a protracted, downwardly rotated, and anteriorly tipped scapular position together with an increased lordosis and upper thoracic kyphosis [12]. This posture brings the origin and insertion of the PMm closer together, creating an environment for a shortened PMm length, which may ultimately result in shoulder impingement [12,13]. Recent local literature has determined pain as one of the problematic SHCs after SCI [2,14–16], however, there is a dearth of literature on the influence of pain in people with SCI in the South African context. This study, to the knowledge of the authors, is the first in South Africa to determine the dynamic interaction of the physical and contextual factors within a pain construct, particularly the interaction between pain, shoulder function, wheelchair functioning, and QOL in PWSCI.

## Research methods and design

### Study design, setting, and population

This study used a quantitative approach and a correlational design. Databases of four consenting rehabilitation hospitals (one private and three government) that admit PWSCI were perused to identify potential manual wheelchair users with paraplegia for participation in the study. To be included in the study, the participants needed to have intact innervation to the shoulder girdle to have an active function of the pectoralis minor muscle and scapular movements, hence limiting to people with paraplegia. Eligible participants needed to be at least six months post-discharge from rehabilitation to be invited to participate. The six-month minimum post-discharge period was chosen as it allowed for potential participants to possibly achieve an optimum level of independence post-discharge from the hospital and for soft tissue changes, such as muscle shortening, to possibly occur with prolonged wheelchair use. A consecutive sampling method was employed and the events per variable (EPV) approach was used to determine the sample size (where EPV > 5) [17]. Vittinghoff & McCulloch [18] support the decision not to discount significant results from 5 EPV and we have corrected for optimism by using bootstrapping in the inferential statistical analyses in this study to derive bias-corrected confidence intervals. A minimum of 35% of manual wheelchair users were anticipated to report pain, with a function of no more than eight variables namely; age, gender, type of occupation, years living with SCI, neurological level of injury, completeness of injury, PMm length, and the presence of scapular dyskinesis. At least 115 participants were required following the sample calculation where EPV > 5, that is, number of events >5 × 8 = 40, and 40/0.35. We excluded consenting participants if they were readmitted to the hospital or moved residences beyond the driving range of a 500 km radius from their discharging hospital at the time of data collection.

### Data collection procedure

Databases of the consenting rehabilitation hospitals were perused from May 2018 to December 2018, and potential participants who were identified were then contacted via telephone to invite them to participate in the study. Those who gave verbal consent were visited by the lead author from February 2019 to March 2020 and an informed consent form was signed on the day of the visit. The lead author is fluent in Sepedi, IsiZulu, and English, and there were no language barriers experienced during data collection.

### Data collection tools

A socio-demographic and injury profile capture sheet was used to document the participants’ demographic and SCI profiles. The painful areas were coded from the most painful to the least painful, namely, the first painful area (P1), until the fifth painful area (P5). Pain severity was determined using the Numeric Rating Scale (NRS), which measures the subjective intensity of pain on an 11-point Likert scale (zero to 10). The NRS has adequate construct and content validity in the SCI population [19]. The Wheelchair Skills Test questionnaire (WST-Q) was used to determine the participants’ wheelchair skills in their daily environment. The WST-Q has high internal consistency reliability α = 0.90, test-retest reliability 0.91 (ICC = 0.84–0.94) high inter-rater reliability 0.86 (ICC = 0.92–0.95), and intra-rater reliability 0.95 [20]. The physical health domain (seven items) and overall QOL items (two items) of the World Health Organization Quality of Life Brief Version (WHOQOL-BREF) questionnaire were used to determine the participant’s QOL and raw total scores were transformed and used to determine the overall QOL score [21]. The WHOQOL-BREF showed high reliability at α = 0.74–0.87, an item-domain validity of *r* = 0.41–0.77, and correlated with the SF-36 at *r* = 0.33–0.78. It also showed excellent inter-rater reliability (ICC = 0.84–0.93) [21]. To determine the presence of pain, the Wheelchair User’s Shoulder Pain Index (WUSPI) was used where the participant answered 15 questions relating to their shoulder pain during activities on a 10 cm visual analogue scale. The total score is summed up from all the item scores (0–150) and higher scores show greater interference of the shoulder pain. The WUSPI has high internal consistency (Cronbach alpha = 0.97) and high test-retest reliability (intraclass correlation coefficient = 0.99) [22]. The WST-Q and the WUSPI are yet to be validated in the South African context.

To measure PMm length, landmarks were identified in the supine position where the coracoid process and the inferior medial aspect of the fourth rib were identified, and the landmarks have an intraclass correlation coefficient (ICC) of 0.96 [23]. Similar to Komati et al. [24], the relaxed, active, and stretched PMm lengths were measured in a supine position with the elbows bent and the hands resting on the abdomen to eliminate the influences of gravity and the passive insufficiency of the biceps brachii muscle on PMm length [9]. The lead author performed all the measurements using a Vernier® caliper and a research assistant recorded the values. A Vernier® caliper was used to minimise the influence of anterior chest wall soft tissue and measurements were done following exhalation to avoid measuring an expanded chest due to inhalation [10,23]. The use of a Vernier® caliper has been shown to have an ICC of 0.83–0.87 [23]. Three measurements were performed and recorded for each position and an average was calculated for a final PMm length. For the resting PMm length, participants were in a relaxed natural posture. The active PMm length was measured with participants performing an active scapula retraction to the point of lumbar extension and the retracted position was sustained during the measurement. The research assistant performed a passive scapular retraction for the stretched PMm length, which was also sustained during the measurement. The participant’s height was measured in a supine position using a retractable measuring tape and the PMm length was expressed using the pectoralis minor index (PMI), calculated as (PMm length(cm)/ height(cm) x 100) [10]. There is no determined threshold of PM shortening as yet, so we followed the recommendation by the literature to rather calculate and express PMI for the sample in each study [10,25]. Lewis and Valentine [26] found the PM muscle length measure to have excellent intra-rater reliability however lacked diagnostic accuracy in the manner in which they tested (using a rigid standard plastic transparent right angle).

To observe a visible alteration in the position of the scapula and scapular motion and determine the presence of scapular dyskinesis, the scapular dyskinesis test (SDT) was used where the participants performed bilateral and active weighted shoulder flexion and abduction for five repetitions each. The SDT classifies the scapular motion as normal, subtle dyskinesis, or obvious dyskinesis [27]. Participants simultaneously elevated their shoulders overhead as far as possible to a three-second count using a ‘thumbs up’ position then lowered their shoulders to a three-second count. The scapular motion was video recorded and 1.4 kg dumbbell weights were used and the weight is determined by the participant’s body weight. We included the use of the dumbbells as an abnormal scapular movement is often visible with resisted active movements than non-resisted movement [27]. McClure et al. [27] confirmed the validity of the SDT with a Kappa reliability of 0.8544 (*p* < 0.001).

### Data analysis

Microsoft Excel (version 2016) was used to manage all collected data and the SPSS v29 was used to analyse the data. Frequencies, percentages, means, standard deviations, and 95% confidence intervals are used to report the descriptive statistics. We performed bootstrap during inferential analyses where the bootstrap results were based on 1000 samples at 95% confidence intervals. Pearson r tests were used to analyse correlations and Fisher’s exact tests to provide information on associations between the variables. Independent t-tests were used to compare two groups (those with and without pain) and one-way analysis of variance (ANOVA) tests were used to examine differences between more than two groups in the case of sleep satisfaction. Testing was done at the 0.05 level of significance and 95% confidence intervals.

### Ethical considerations

This study is registered with the South African National Health Research Database (reference number GP201806005) and was approved by the Faculty of Health Sciences Research Ethics Committee, University of Pretoria (approval number 125/2018). Furthermore, permission has been granted by the participating rehabilitation hospitals, and written informed consent was obtained from all the participants in this study. There were no financial costs or financial compensation involved for the participants in this study. All data from this study is stored at the University of Pretoria’s Physiotherapy Department for no less than fifteen years as per the data safekeeping requirements of the research ethics committee.

### Control of bias

To reduce the risks of recall bias, the researcher limited recall to four weeks, as per the guidelines of the WHOQOL-BREF [21]. To minimize selection bias, all eligible participants from the four databases were contacted to invite them to participate and the study included all those who consented. To reduce the Hawthorne Effect [28], participants were told that there is no wrong or right answer and that the study aimed to determine how their pain may or may not influence their general health satisfaction and how they use their wheelchairs. Although all questionnaires used in this study are self-reported, the lead author administered them to ensure consistency in the way the questions were understood and to minimise measurement errors. The lead author also undertook sessions with the second author to ensure that the correct PMm landmarks were identified and measured, as well as sessions with an external expert to undergo the correct identification of scapular motion.

## Results

### Socio-demographic and injury profile

A total of 122 PWSCI participated in the study and the mean age of participants in this study was 39.7 years (SD 11.1) and the mean age when injured was 32.6 years (SD 10.7), while the mean years living with SCI was 7.1 years (SD 7.1). Most of the participants were male (68.0%); resided with their family (50.8%); were unemployed (59.0%); were on the government disability grant (47.5%); and did not have other health problems (80.3%). The aetiology of SCI was mostly traumatic SCI (85.2%), with motor vehicle accidents as the most common cause of SCI (41.0%). Complete SCI was more common (76.2%) and most of the participants had neurological levels of injury between T6-T12 (73.8%).

### Overall pain presentation

The majority of the participants experienced current pain (85%) which was experienced mainly in one area of the body (48.4%), 27.9% in two areas, 5.7% in three areas, 2.5% in four areas, and 0.8% in five areas. The mean pain severity for the most intense pain (P1) was 6.7 (SD 2.3), 2.4 for P2 (SD 3.0), 0.6 for P3 (SD 1.7), and 0.2 for P4 (SD 0.9). The higher standard deviations more than their means for P2, P3, and P4 respectively, suggest a large variability and analysis confirmed the skewness of P3 (2.98) and P4 (6.92). Only one participant (1%) reported P5 as muscular aches located in the hands, with a severity of 3.5 out of 10. Pain occurred throughout the body, however, the lower limbs below the injury level were the most common location of pain experienced (39.4%). The most common behaviour of pain was described as burning (32.7%).

### Shoulder pain

Only 18 participants (14.8%) reported shoulder pain and all of them experienced pain in their dominant shoulder. Two participants with shoulder pain reported previous shoulder operations, namely, an internal fixation following a shoulder dislocation; and a rotator cuff repair. The WUSPI mean score was 63.27 (SD 35.11), with pushing a wheelchair up inclines being the most painful (Table 1) and a 42.5% shoulder pain interference.

**Table 1:** WUSPI descriptive statistics (n = 18).

WUSPI items	Mean	Std. Deviation
Transfer from bed to wheelchair	3.94	2.920
Transfer from wheelchair to car	4.31	3.295
Transfer from a wheelchair to tub or shower	5.20	3.084
Loading your wheelchair into car	4.00	2.366
Pushing your chair for ten minutes or more	4.81	2.750
Pushing up ramps or inclines outdoors	6.06	3.269
Lifting objects down from an overhead shelf	5.29	3.405
Putting on pants	3.00	3.678
Putting on a t-shirt or pullover	3.00	3.926
Putting on a button down shirt	1.29	2.592
Washing your back	4.40	2.694
Usual daily activities at work or school	4.83	3.382
Driving	3.38	3.623
Performing household chores	4.13	3.096
Sleeping	4.94	3.796
Total	63.72	35.11

### Shoulder biomechanics

a) Pectoralis minor muscle lengths

Our participants had a mean resting PM muscle length of 17.72 cm (SD 2.36) on the dominant side and 19.18 cm (SD 14.11) on the non-dominant side. The PMm length means, expressed as PMI, are included in Table 2.

**Table 2:** Pectoralis minor index descriptive statistics.

Pectoralis minor muscle position		Those with shoulder pain (n = 18)		Those without shoulder pain (n = 86)	
		Mean	Std. Dev.	Mean	Std. Dev.
Dominant shoulder	Relaxed	10.80	0.93	10.51	1.49
	Active	11.13	0.85	10.79	1.52
	Stretched	11.93	1.06	11.74	1.64
Non-dominant shoulder	Relaxed	10.93	0.90	10.65	1.48
	Active	11.31	0.77	11.01	1.54
	Stretched	12.16	1.01	11.88	1.65

We found no significant differences in PMI between those with and without pain, irrespective of whether the PM muscle was in its relaxed, active, or stretched positions. We also did not find any significant association between shoulder pain and PMI.

b) Scapular motion

Five participants, who did not report any shoulder pain, did not consent to the video recording required for the SDT to determine the scapular motion. Four participants with shoulder pain and 17 without shoulder pain were unable to maintain the necessary balance required for the SDT. Table 3 illustrates the proportions of the participants with and without pain against the SDT results. We found no significant association between scapular dyskinesis and shoulder pain (Fisher’s exact = 1.15, p = 0.944).

**Table 3:** The proportion of participants with and without shoulder pain when testing their scapular motion.

Description of the SDT	Those without shoulder pain (n = 82)	Those with shoulder pain (n = 14)	Total
Normal scapular motion (Proportion in %)	5	1	6
	83.3%	16.7%	
Subtle scapular dyskinesis (Proportion in %)	3	0	3
	100.0%	0.0%	
Obvious scapular dyskinesis (Proportion in %)	74	13	87
	85.1%	14.9%	

### Pain and wheelchair function

Four participants only used their manual wheelchairs in new areas or for long distances and mainly used walking frames or crutches for indoor mobility, of these, two had reported overall pain. The total number of participants assessed for wheelchair function is therefore 118, with 18 reporting specific shoulder pain and 16 without any pain. We found an overall WST-Q score of 48.28% (SD 10.25), and 48.76 (SD 10.32) for those with pain (n = 102) (Table 4). Table 4 also depicts the different responses of those with shoulder pain (n = 18). There was no difference in wheelchair function found between participants with and without pain (*t* = −1.034, df = 116, *p* = 0.195), irrespective of whether the pain was nociceptive or neuropathic (*t* = 0.451, df = 100, *p* = 0.653).

**Table 4:** Wheelchair Skills Test Questionnaire descriptive statistics between those with and without pain only.

		Those with pain (n = 102)			*Only those with shoulder pain (n = 18)*			Those without any pain (n = 16)		
		Capacity			*Capacity*			Capacity		
#	Individual Skill	0	1	2	*0*	*1*	*2*	0	1	2
1	Rolls forwards short distance	0	0	102	*0*	*0*	*18*	0	0	16
2	Rolls backwards short distance	0	0	102	*0*	*0*	*18*	0	0	16
3	Turns while moving forwards	0	1	101	*0*	*1*	*17*	0	0	16
4	Turns while moving backwards	0	0	102	*0*	*0*	*18*	0	0	16
5	Turns in place	0	1	101	*0*	*1*	*17*	0	0	16
6	Maneuvers sideways	1	3	98	*0*	*1*	*17*	1	0	15
7	Gets through hinged door	2	3	97	*0*	*2*	*16*	1	1	14
8	Reaches high object	3	9	90	*0*	*4*	*14*	3	1	12
9	Picks object from floor	3	4	95	*1*	*1*	*16*	1	1	14
10	Relieves weight from buttocks	1	2	99	*0*	*0*	*7*	1	0	15
11	Transfers to and from bench	2	2	98	*0*	*2*	*16*	3	0	13
12	Folds and unfolds wheelchair	33	19	50	*8*	*3*	*7*	7	2	7
13	Rolls longer distance	3	12	87	*0*	*3*	*15*	2	2	12
14	Avoids moving obstacles	2	0	100	*0*	*0*	*18*	0	0	16
15	Ascends slight incline	3	12	87	*0*	*5*	*13*	1	2	13
16	Descends slight incline	3	11	88	*0*	*4*	*14*	1	2	13
17	Ascends steep incline	17	44	41	*3*	*10*	*5*	3	5	8
18	Descends steep incline	18	41	43	*3*	*9*	*6*	3	4	9
19	Rolls across side-slope	16	30	56	*4*	*8*	*6*	4	2	10
20	Rolls on soft surface	6	21	75	*1*	*6*	*11*	1	2	13
21	Gets over gap	4	6	92	*1*	*2*	*15*	2	1	13
22	Gets over threshold	2	4	96	*0*	*0*	*18*	0	1	15
23	Ascends low curb	17	12	73	*7*	*2*	*9*	4	4	8
24	Descends low curb	17	12	73	*7*	*2*	*9*	4	3	9
25	Ascends high curb	43	47	12	*13*	*4*	*1*	10	5	1
26	Descends high curb	43	44	15	*13*	*4*	*1*	10	5	1
27	Performs stationary wheelie	56	2	44	*10*	*0*	*8*	11	0	5
28	Turns in place in wheelie position	75	0	27	*13*	*0*	*5*	14	0	2
29	Descends steep incline in wheelie position	78	1	23	*13*	*0*	*5*	14	1	1
30	Descends high curb in wheelie position	82	2	18	*13*	*1*	*4*	14	1	1
31	Gets from ground into wheelchair	71	8	23	*15*	*0*	*3*	13	1	2
32	Descends stairs	98	2	2	*16*	*1*	*1*	15	1	0
Total		48.76 (SD 10.31)			*45.89 (SD 0.89)*			45.18 (SD 9.49)		

When investigating wheelchair function and shoulder pain, we found no significant differences between those with and without shoulder pain. However, there was a negative correlation between shoulder pain and wheelchair function in those who reported shoulder pain (*r* = −0.606, *p* = 0.008).

Wheelchair function yielded no significant correlation to pain severity in participants who experienced pain (*r* = − 0.104, *p* = 0.299). We, however, found a generally low and negative correlation between age and the total WST-Q score (*r* = − 0.331, *p* < 0.001), which may suggest that the ability to perform wheelchair skills decreases as age increases. Overall WST-Q scores yielded low correlations with overall QOL scores (*r* = 0.298, *p* = 0.002), which may suggest that PWSCI have good QOL when they have good wheelchair skills. When analysing wheelchair function with specific QOL items, we did not find any significant difference between participants with and without pain and the ability to get around using their wheelchair, nor with their satisfaction with the way they executed their ADLs. This lack of difference was confirmed with a one-way ANOVA test [F (5,112) = 1.294, *p* = 0.272 and F (4,113) = 1.791, *p* = 0.135 respectively].

### Overall pain and quality of life

The overall mean QOL score was 62.3% (SD 17.93), with a mean of 61.23 (SD 17.42) for those with pain and 68.67 (SD 19.98) for those without pain. There was no difference in QOL between participants with and without pain (*t* = 1.636, *p* = 0.105), irrespective of whether the pain was nociceptive or neuropathic (*t* = 1.383, *p* = 0.170).

Despite pain not impacting overall QOL, a large proportion of participants (72%) who experienced pain reported that the pain ‘extremely’ prevented them from doing what they needed to do (Fisher’s exact = 11.227, *p* = 0.013). The Fisher’s exact test also showed a significant association between the presence of pain and health satisfaction (Fisher’s exact = 11.786, *p* = 0.021). Ninety-seven percent of participants were neither satisfied nor dissatisfied with their health.

Participants with increased pain levels were less satisfied with their sleep [F (4,99) = 3.328, *p* = 0.013]. Post-hoc least significant difference (LSD) tests revealed pain severity was statistically significantly lower in participants who were satisfied with their sleep (M = 5.94, SD = 2.2 with a mean difference of 1.98 points on the NRS) and very satisfied with their sleep (M = 6.19, SD = 2.37 with a mean difference of 1.73) compared to those who were dissatisfied with their sleep (M = 7.92, SD = 2.02).

We also found a moderate and negative correlation between the severity of pain and QOL in people who reported pain (*r* = − 0.411, *p* < 0.001), suggesting that QOL decreases as pain severity increases.

## Discussion

The high presence of overall pain in our study is unsurprising, given that pain after SCI is common [1,3,29], and PWSCI who experience pain are at an increased risk of poorer functioning as compared to those without pain [29]. Our findings of burning type of pain below the level of injury being more common is similar to the literature [30]. However, our shoulder pain rates are low as compared to the literature. For example, Rafiullah et al. [31] and Bossuyt et al. [32] found 34.7% and 35.8% of shoulder pain in their SCI populations respectively. Nociceptive pain after SCI is commonly a result of musculoskeletal overuse injuries during activities [33] and our population was mainly unemployed, suggesting that they may have not been participating in strenuous and over-exertive daily activities beyond the minimal wheelchair propulsion and transfers. There are many possible causes of shoulder pain over and above over-exertion from wheelchair use, and a thorough assessment is necessary to determine the root cause of the shoulder pain. Musculoskeletal pain may also be due to muscle imbalances, sustained abnormal postures, or movements associated with muscle weakness in the upper limbs [33]. People with tetraplegia have poorer shoulder musculoskeletal integrity when compared to those with paraplegia due to muscle imbalances and limited range of movement in the shoulder joint owing to the partial loss of motor function in the shoulder girdle [34]. Furthermore, people with tetraplegia have up to 1.82 higher odds of shoulder pain than those with paraplegia [32], which may explain our low shoulder pain rates in this population of people with paraplegia.

The high pain severity for the most intense pain is consistent with available literature and underpins the seriousness of pain as a problem after SCI [35]. Similar to the literature, we found that pain intensity is associated with interference in activities, and the more severe the pain, the more substantial the negative impact on very basic activities such as sleep, and ultimately QOL [1]. We found a moderate level of QOL in our study for those with and without pain, similar to reports by Khazaeipour et al. [6]. Pain is reported to negatively affect general well-being [36] and we found a high number of participants who were indifferent about their health and reported to be neither satisfied nor dissatisfied with their health.

The low scores of wheelchair functioning found in our study from people who use manual wheelchairs are concerning. Wheelchair propulsion is not easy, and Vincent et al. [37] reported the activity as challenging, and worse when advanced wheelchair skills such as uneven terrains, prolonged propulsion, and inclines are considered. This does not bode well together with the negative relationship we found between wheelchair function, QOL, and age. Our findings suggest that PWSCI have good QOL when they have good wheelchair skills, but that the ability to perform wheelchair skills decreases as a person with SCI ages. Wheelchair mobility is not only fundamental in life after SCI, it is necessary for independence and has been shown to have a positive impact on social well-being, physical fitness, and overall QOL [38]. Wheelchair skills may be quickly improved with minimal training and practice following proper education [38] and should be considered to ultimately improve QOL and maintain the skill across the lifespan of PWSCI. Pain after SCI is a burdensome SHC and one must note the predisposition of shoulder pain with prolonged wheelchair use, either due to overuse or misuse injuries [3,13].

Although this study found that pain prevents PWSCI from doing what they need to do, this does not seem to include wheelchair functioning as no relationship was found between decreased wheelchair function and pain. This is not surprising, as PWSCI are known not to rest their shoulders even in the presence of shoulder pain [2]. This may also explain why we found no difference in wheelchair function between those with and without pain. People with SCI depend on their upper limbs for mobility and transfers and resting their shoulders may prove a challenge as supported by Eriks-Hoogland et al. [39]. Dependency on wheelchair use is known to predispose PWSCI to shoulder pain due to the repetitive loading of the shoulder joint [31,40]. Although increased wheelchair use has been linked with shortened PMm length and shoulder pain from overuse injuries, we did not find the pain to be associated with PMm muscle length nor the presence of scapular dyskinesis, similar to findings by Finley and Ebaugh [13]. Our findings suggest that biomechanical changes in the shoulder are habitual following SCI, not due to the presence of pain. Despite common expectations, from the above literature, this study also did not find any associations between PMm length and the presence of scapular dyskinesis with wheelchair function. The majority of the participants in the current study had shortened PMm length and obvious scapular dyskinesis even without pain, which is understandable due to the shoulder and scapular demands that come with wheelchair use. Wheelchair propulsion causes some muscles to work more than others (such as the shoulder flexors, internal rotators, and adductors) causing muscle imbalances or changes in the muscle activations, and ultimately resulting in scapular dyskinesis [27]. Our findings therefore suggest that scapular dyskinesis in manual wheelchair users may need further assessment for causative factors, as supported by Sciascia and Kibler [41].

This study did not find a significant difference in QOL between people with and without pain, suggesting that overall QOL in this population was related to the SCI itself and not exclusively to pain, similar to findings by Rivers et al. [42]. Furthermore, the presence of pain extremely prevented PWSCI from doing what they needed to do and they were neutral with regards to their health satisfaction. Even though pain (whether nociceptive or neuropathic) did not impact wheelchair function in this study, a concerning finding was that wheelchair function decreases with age, especially as it was also found that good wheelchair function was positively associated with increased QOL. This suggests that as PWSCI age, their wheelchair skills will diminish – owing to old age – and their QOL will subsequently decrease as a result. The upper limbs absorb increased load during wheelchair activities such as transfers, and the persistent stress on the acromioclavicular joint may lead to joint degeneration, which has been found in 28.5% of people with paraplegia [43]. The natural aging process may further exacerbate the joint degeneration in PWSCI. Modifications to reduce wear and tear on the shoulders (such as using a transfer board to transfer in and out of bed or the car) are therefore necessary to preserve the shoulder joint and prevent premature degeneration of the already vulnerable shoulder joints of PWSCI.

## Strengths and limitations

This is the first study to the knowledge of the authors that determined the correlation between pain, wheelchair functioning, quality of life, and shoulder biomechanics in the South African context. Community-dwelling manual wheelchair users across four provinces were visited. By delimiting the study to those with paraplegia only, the authors could determine musculoskeletal shoulder pain that is not related to loss of innervation to the upper extremities because of cervical SCIs in the case of those with tetraplegia. However, only including those with paraplegia limits generalisation to those with tetraplegia. Furthermore, our study participants were recruited from rehabilitation units in Gauteng, and generalisation should be done under consideration. This study was not able to measure the participant’s body weight to determine the dumbbell weight as specialised equipment, which is not suitable for traveling, would be required. Unlike McClure et al. [27] who used a 1.4 kg dumbbell weight for able-bodied participants who weighed less than 68.1 kg and 2.3 kg for those weighing 68.1 kg or more, we rather used 1.4 kg weights for all our participants due to the dynamic sitting balance challenges that are present after a SCI. Furthermore, this study did not classify the participants according to the American Spinal Injury Association (ASIA) Impairment scale, which may have influenced the reporting of the below-level pain that was reported. The location of the neuropathic pain may have been at-level, and not below-level. This study also did not explore the interaction between the completeness of injury and the below-level pain that was reported, which may have been beneficial in understanding the interaction of pain and functioning.

## Conclusion

This study underscores available findings that pain is a problematic SHC in PWSCI. Pain prevents PWSCI from doing what they need to do and they are not optimally satisfied with their health. Increased severity of pain not only reduces sleep satisfaction but is negatively correlated with QOL. Future studies are necessary to reduce the severity of pain and improve overall wheelchair function to increase the potential of an improved QOL in a person with SCI.
